# The International Medical Congress

**Published:** 1900-08-11

**Authors:** 


### The International Medical Congress.

If one regards the events in midst of which we have been living—the monstrous and abominable assassination of the King of Italy, the attempt on the life of the Shah, the death of an honoured member of our own Royal Family, the anxious doubt about South African affairs, the critical situation in China and the constant possibility of international complications of untold gravity arising—it cannot be said that the International Medical Congress met last week in Paris under the happiest auspices. On the other hand, its physical and material surroundings were of the brightest. Instead of being the stewpan it so often is in August, Paris, if not at its best, was at least in its most exhilarating phase, bright with sunshine, cool with breezy air, and with now and again a shower to keep down the dust—just what was best for a great meeting, and most conducive to that interchange of thought which is of the very essence of a congress; and if an air of holiday-making were pretty obvious, and if under the distraction of science and pleasure-seeking one was able for a time to put aside the newspaper and all sombre politics, one must be forgiven.

The opening meeting of the Congress was held in the *Salle des fêtes* in the Exhibition. A platform had been erected for the honorary presidents of the Congress, for the officials, the delegates of the foreign States, the presidents of the sections, the representatives of the Institute, the Academies, the Faculty, &c., and in front of this platform spread a vast sea of faces—in front the delegates of the various public bodies, universities, scientific societies, &c., and behind them the ordinary congressists—to use the word that has been invented to describe the members of a congress; while far away at the other side of the vast hall was the tribune reserved for the ladies, who, however, by no means reserved themselves to their tribune, but showed their good sense by permeating the whole meeting, the platform itself being, so far as we could see, the only place sacred to the nobler sex.

Great, however, as was the assembly, the room in which it was held was vaster still, with the result that much that went on upon the platform was a mere matter of dumb show. A figure popped up—sometimes in plain costume, sometimes resplendent with sash and order, sometimes brilliant with medals and decorations; a sudden thunder or a gentle ripple of applause according to the nationality of the figure arose in the neighbourhood, the thunder usually meaning that a German was on his legs, for they held together bravely—and then for five minutes came the monotonous sound of distant oratory, its point of origin being marked by a gesticulating figure, and so on for an hour or two.

Clearly for ceremonial purposes it was a great mistake to have so large a room, for directly those in the background found they were out of it they walked about and chatted with their friends, and laughed and generally enjoyed themselves, and so still further narrowed the circle in which hearing was possible.

The President of the Republic was to have opened the Congress in person, but Professor Lannelongue, the President of the Congress, informed the assembly that in consequence of the crime committed on the King of Italy the President had decided not to take part in any official ceremony. M. Lannelongue expressed to our Italian confrères the sympathy felt for them by French physicians under the circumstances, and then proceeded to deliver his address, urging upon the Congress the necessity, in view of the extremely embarrassing nature of the problems involved in all medical investigations, that the fullest use should be made of instruments of precision so as to secure accuracy of observation, and that accuracy of record should be secured by employing a form of nomenclature far more in accordance with the present state of our knowledge, and much more capable of universal acceptance in all countries than any that has so far been employed. Finally, he heartily welcomed the congressists to Paris.

M. Virchow then read a paper on Traumatisme, discussing the various theories which have been held as to the cause of the inflammatory and other changes which sometimes supervene upon injuries of the deeper parts where the skin has remained unbroken, and where direct infection from without would seem improbable.

Friday and Saturday were devoted to the various sections, which threaten to carry on with unabated vigour far into this week their search and their dissemination of knowledge. Much, however, is being done besides mere section work; hospitals are being visited, receptions are to be held, excursions to places of medical interest are to be made, nor while the mind is being enlightened is the body neglected, for much hospitality is going on besides what appears 'on the bills." In every way the International Congress is going to be successful, for if one tires of science and "sections," and of talk in various tongues, there is always the Exhibition to turn into, and after, or perhaps even before the Exhibition, is there not always Paris?

